# Overlapping Spectrum of Impulsivity and Compulsivity Across Psychiatric Disorders: A Narrative Review on Dimensional Perspectives

**DOI:** 10.5152/eurasianjmed.2025.24749

**Published:** 2025-06-20

**Authors:** Ali Kandeğer

**Affiliations:** Department of Psychiatry, Selçuk University Faculty of Medicine, Konya, Türkiye

**Keywords:** ADHD, compulsivity, impulsivity, obsessive compulsive disorder, personality disorders

## Abstract

Impulsivity and compulsivity are complex constructs that underpin a wide range of psychiatric disorders. While traditionally viewed as opposing dimensions, emerging evidence suggests they exist on an overlapping spectrum, influencing diagnosis and treatment. Impulsivity is characterized by poorly planned, premature responses aimed at achieving gratification, while compulsivity involves repetitive behaviors driven by anxiety relief. Both constructs share common neurobiological substrates, such as impaired response inhibition and urgency, but differ in their executive dysfunction patterns. Psychiatric disorders traditionally associated with impulsivity (e.g., attention-deficit hyperactivity disorder, bipolar disorder) and those aligned with compulsivity (e.g., obsessive-compulsive disorder, anorexia nervosa) often exhibit overlapping symptoms. Additionally, the same disorder may present varying levels of impulsivity and compulsivity across individuals and time points. The impulsivity-compulsivity spectrum offers a dimensional framework for understanding psychiatric disorders, emphasizing the need for individualized clinical approaches. This review aims to highlight the shared and distinct features, as well as the neurobiological pathways, associated with this spectrum, while underscoring the need for future research to refine dimensional models that enhance diagnostic accuracy and guide personalized interventions.

**Main Points**

- Both impulsivity and compulsivity reflect difficulties in perseverance: impulsivity is driven by an urgent need for pleasure, and compulsivity is driven by an urgent need to relieve distress. In both cases, the individual seeks rapid change in their emotional state.
- Impulsive decision-making is driven by an overactive reward system, with studies linking immediate reward choices to heightened activity in the ventral striatum and medial prefrontal cortex.
- Compulsive behavior involves dysfunction in fronto-striatal-thalamo-cortical pathways, linking repetitive actions to altered orbitofrontal and habit-forming circuit activity.
- Despite differences, both constructs suggest dysfunctions in the top-down inhibition of behavior through different neural circuits.
- Research is yet to identify a psychiatric disorder driven purely by either impulsivity or compulsivity, underscoring the intertwined and overlapping nature of these 2 constructs.

## Introduction

The term impulsivity encompasses responses and behaviors to internal or external stimuli that are often premature, insufficiently planned, and therefore frequently inappropriate and immature.^1^ Before considering impulsivity as a psychiatric symptom, it is essential to remember its biopsychosocial foundation and its close relationship with certain temperament and personality dimensions. For example, in Eysenck’s 3-factor personality model, impulsivity is included under the psychoticism and venturesomeness dimensions, while sensation-seeking is associated with the extraversion dimension.^2^ Similarly, Cloninger’s temperament and character model incorporates novelty-seeking, defined as a tendency to prefer acting on emotions rather than adhering to momentary rules or regulations, under the novelty-seeking dimension.^3^ As with many symptoms considered diagnostic criteria in psychiatric disorders, it is crucial to distinguish whether what appears as impulsivity is a functional impairment or simply a temperament or personality trait.

In clinical practice, impulsivity is considered a core symptom of some psychiatric disorders and a diagnostic criterion for others.^1^ Clinicians are more likely to recognize impulsivity as a prominent feature in disorders such as attention-deficit hyperactivity disorder (ADHD), impulse control disorders, Cluster B personality disorders, and bipolar disorder. This is because the fundamental characteristics of impulsive responses—quick, unplanned, and made without consideration of consequences—are relatively easy to identify. However, defining the boundaries of impulsivity is more complex. This complexity relates to understanding behavioral patterns that, when questioned, may be described as habits but tend to repeat and impair functionality due to their negative consequences. These patterns have recently been conceptualized within the impulsivity and compulsivity spectrum. Compulsive behaviors, in contrast, are repetitive, unplanned, and stereotyped actions that help individuals with heightened threat perception and increased distress reduce their anxiety, often driven by an overactive harm-avoidance motivation.^4^ Obsessive-compulsive disorder (OCD) is widely recognized as the prototypical disorder representing the compulsive end of this spectrum.^5^

This spectrum conceptualization should not imply that impulsivity is absent in OCD or that compulsive behaviors cannot co-occur in ADHD. Rather, it reflects the core symptoms that define the extremes of these disorders. Importantly, symptoms from both ends of the spectrum are often present in a range of psychiatric conditions. The placement of psychiatric disorders along this spectrum is an ongoing topic of debate. A significant outcome of these discussions has been the reclassification of certain disorders. For example, trichotillomania (hair-pulling disorder), previously classified under impulse control disorders, and body dysmorphic disorder, previously categorized as a somatoform disorder, were included under the OCD-related disorders category in the fifth edition of the Diagnostic and Statistical Manual of Mental Disorders (DSM-5).^6^

This review will examine impulsivity and compulsivity across various psychiatric disorders, exploring their shared and distinct features, and highlighting the dimensional perspective as a critical framework for understanding these constructs. By focusing on disorders such as ADHD, OCD, bipolar disorder, and substance-related conditions, this article aims to elucidate how impulsivity and compulsivity interact within clinical presentations and their implications for diagnosis and treatment.

## Distinguishing Features of Impulsivity and Compulsivity

Research to date has conceptualized impulsivity and compulsivity as 2 distinct dimensions, highlighting their key differences as follows: While impulsive actions are unplanned and spontaneous, compulsive behaviors are accompanied by ruminative, persistent, and rigid thoughts.^7^ Individuals exhibiting compulsive behaviors are more likely to recognize these actions as maladaptive and tend to perceive them as ego-dystonic, meaning they feel alien or contrary to their self-perception.^8^ In contrast, impulsive actions are primarily aimed at seeking pleasure and satisfaction, whereas compulsive behaviors are intended to reduce distress and negative emotions.^1^ This distinction may be the most critical differentiation between the 2 constructs. For instance, gambling driven by the pursuit of excitement and pleasure despite potential negative consequences reflects impulsive behavior. On the other hand, gambling or shopping as a means of alleviating negative emotions aligns more closely with compulsive behavior. Compulsivity is associated with heightened risk perception, whereas impulsivity is linked to the undervaluation of risk.

**Figure 1.**
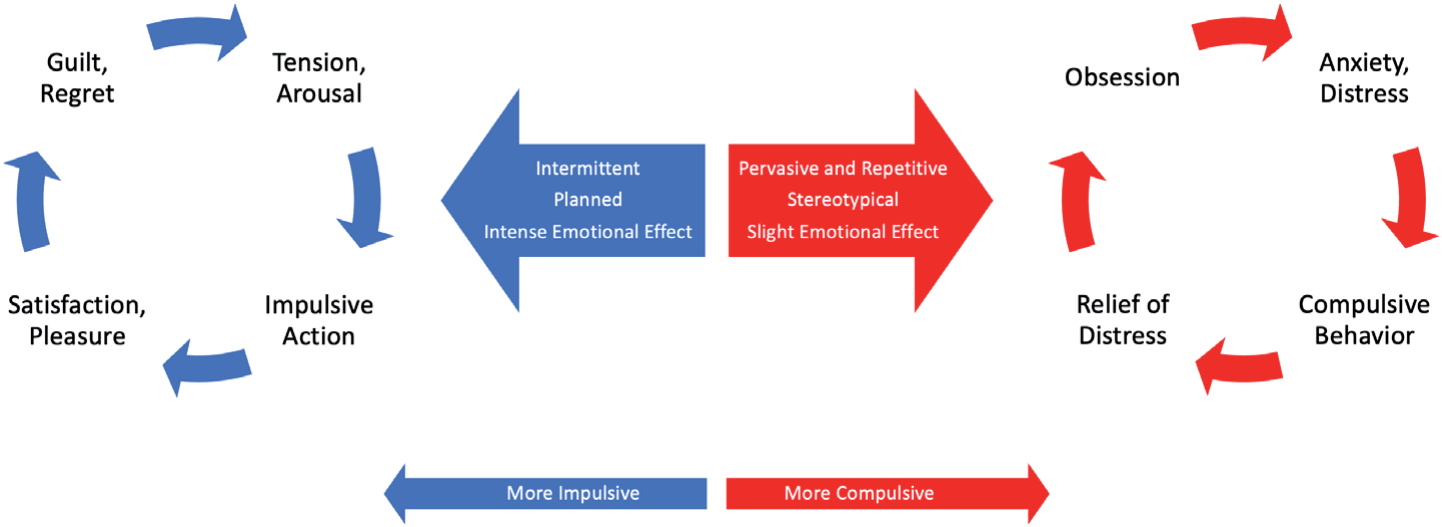
Reinforcement cycles of impulsive and compulsive behaviors.

Lastly, on the impulsivity-compulsivity spectrum, the most impulsive actions are characterized by higher intensity, destructive consequences, and infrequent occurrence, such as days or weeks apart. In contrast, the most compulsive behaviors tend to be less impactful per instance but are stereotypical, repetitive, and pervasive throughout the day, significantly impairing the individual. For example, a person with pyromania may set fires only twice a year, yet the act’s intensity, destructiveness, and consequences are sufficient to disrupt functioning. Conversely, a person with trichotillomania may engage in hair-pulling behavior for hours daily, where the repetitive and stereotypical nature of the behavior underpins its impairing effects. The distinguishing characteristics of impulsive and compulsive behaviors and their reinforcement cycles are schematized in Figure 1.

Differences in executive functions are also evident in impulsive and compulsive behaviors. Executive dysfunctions associated with impulsivity can be categorized into 3 main areas. First, motor impulsivity involves difficulties in suppressing prepotent, non-dominant stimuli following a primary cue requiring action. Individuals with high impulsivity struggle to inhibit themselves when faced with such stimuli. Second, reward impulsivity reflects an inability to delay gratification, with a preference for smaller, immediate rewards over larger, delayed ones. Finally, reflection impulsivity refers to a tendency to make decisions hastily, with minimal information or deliberation.^9^ In contrast, the executive dysfunction most commonly associated with compulsivity involves difficulties in set-switching. This impairment, characterized by an inability to shift attention, leads to perseverative and ruminative patterns that perpetuate compulsive behaviors.^10^

## Neurobiological Pathways of Impulsivity and Compulsivity

Monoaminergic alterations are pivotal in the etiopathogenesis of the impulsivity-compulsivity spectrum. While dopamine dysregulation is closely linked to reward-seeking and impulsive decision-making, serotonin pathways are often implicated in anxiety relief and repetitive, compulsive behaviors.^8^ This distinction further underscores why impulsivity and compulsivity, though overlapping, can manifest through distinct neurobiological processes.

Beyond the monoaminergic hypothesis, cortical and subcortical brain structures, as well as neural circuits, play significant roles in the neurobiological underpinnings of impulsivity and compulsivity.^11^ One of the fundamental components of impulsive decision-making is an overactive reward drive, which has been associated with increased activity in the ventral striatum and medial prefrontal cortex. Previous studies have suggested that choices favoring immediate rewards are linked to disproportionate signal increases in the ventral striatum, medial prefrontal cortex, and medial orbitofrontal cortex. The right inferior frontal gyrus and its associated networks may also play a crucial role in top-down response control, as functional impairments in this area have been associated with impulsive actions.^12^ Thus, distinct but interrelated neural circuits may contribute to various facets of impulsivity. One such circuit can be characterized as a ventral striatal loop, including the ventromedial prefrontal cortex, subgenual cingulate cortex, and nucleus accumbens/ventral striatum.^13^

In contrast, the circuits implicated in compulsivity include reversal learning circuits (dorsolateral prefrontal cortex, lateral orbitofrontal cortex, and caudate nucleus) and habit learning circuits (supplementary motor area, premotor cortex, and putamen). Neurobiological models of OCD propose that dysfunction within fronto-striatal-thalamo-cortical circuits, involving abnormalities in the anterior cingulate cortex, orbitofrontal cortex, thalamus, and basal ganglia, contributes to its pathogenesis.^14^ A shift in the balance of direct and indirect pathways involving distinct basal ganglia pairs—favoring the direct pathway—leads to hyperactivity in the orbitofrontal-subcortical circuit, resulting in compulsive behaviors.^15^ Functional neuroimaging studies have shown increased brain activity and altered functional connectivity in the anterior cingulate cortex in individuals with OCD when symptoms are provoked.^16^

## The Overlapping Nature of Impulsivity and Compulsivity

Traditional expert opinions have primarily regarded impulsivity and compulsivity as 2 distinct and independent constructs.^5^ However, a growing body of scientific research supports the idea that these 2 concepts are partially overlapping, can co-occur, and should be evaluated together in terms of symptom severity. Impulsivity and compulsivity may manifest simultaneously in the same psychiatric disorder, at different times within the same disorder, or only in some individuals meeting specific diagnostic criteria.

While positive reinforcement (seeking pleasure and excitement) is more closely associated with impulsivity, and negative reinforcement (avoiding harm and alleviating distress) is more aligned with compulsivity, the distinction between the 2 can blur when a reinforcing and repetitive behavior persists.^17^ For instance, someone who initially smokes for pleasure may eventually transition to regular use at specific times and places to alleviate withdrawal symptoms. In this case, a process that began as an impulsive act evolves into a compulsive, ritualistic behavior.^9^ Similarly, although planned and ritualized behavior is often linked to compulsivity, individuals with gambling disorder or pyromania may plan their actions to some extent and repeat them at regular intervals. Furthermore, these pleasure-driven behaviors may also arise as a way to regulate tension and distress following an increase in negative emotions.^17^

Both impulsivity and compulsivity can be understood as issues of persistence. In impulsivity, there is an urgency driven by a desire to quickly achieve pleasure, while in compulsivity, the urgency is tied to a rapid need to reduce distress. In both cases, the individual seeks an immediate change in their emotional state. As a result, both constructs involve dysfunctions in the top-down control of behavior through different neural circuits.^12^ Behavior or response inhibition is a key target in the cognitive-behavioral techniques proposed for managing both constructs. The inhibition of pleasure-seeking behaviors in impulsive actions and distress-alleviating behaviors in compulsive patterns aims to extinguish the challenging emotions driving these behaviors.^18^

This can be further clarified through the widely accepted 5-dimensional model of impulsivity, which includes the following constructs: positive urgency (acting hastily under positive emotions), negative urgency (acting hastily under negative emotions), lack of premeditation, lack of perseverance, and sensation-seeking.^19^ Among these dimensions, “negative urgency” and “lack of perseverance” are also characteristics described in the definition of compulsive behaviors. Moreover, given that compulsive behaviors are often unplanned and stereotypical, “lack of premeditation” could also be associated with compulsivity. This suggests that even the methods for assessing impulsivity and compulsivity struggle to fully separate these constructs.

**Figure 2.**
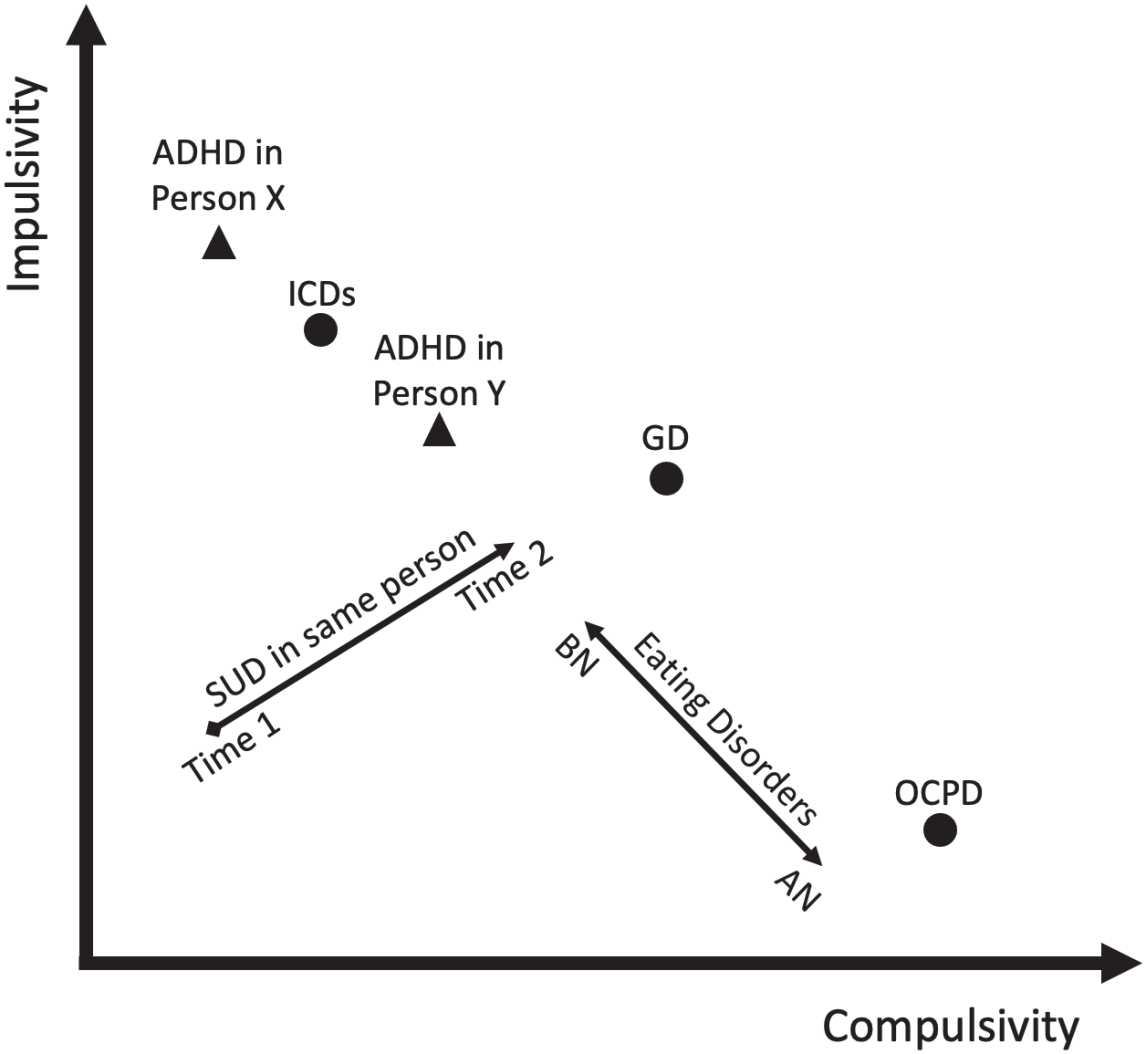
Psychiatric disorders along the impulsivity-compulsivity spectrum. The impulsivity-compulsivity spectrum can be visualized as an X-Y axis, with disorders positioned at various points rather than strictly at opposing poles. While ICDs cluster near impulsivity and OCPD aligns with compulsivity, some conditions (e.g., GD) display both features. As illustrated by ADHD and SUD, the same diagnosis—or even the same individual—can shift along the spectrum over time. Within EDs, AN tends toward compulsivity, whereas BN leans more toward impulsivity. *Abbreviations:* ICD = Impulse Control Disorder; OCPD = Obsessive-Compulsive Personality Disorder; GD = Gambling Disorder; ADHD = Attention Deficit Hyperactivity Disorder; SUD = Substance Use Disorder; ED = Eating Disorder; AN = Anorexia Nervosa; BN = Bulimia Nervosa.

In line with this, DSM-5 reclassified kleptomania and other body-focused repetitive behaviors (BFRBs, such as skin-picking, nail-biting, and lip-biting) from impulse control disorders to OCD-related disorders. Furthermore, consistent findings indicate that individuals with OCD exhibit higher levels of impulsivity compared to healthy controls. This has led to the hypothesis that OCD may not be confined solely to anxiety-avoidance behaviors but could also represent a form of behavioral addiction.^20^ Evidently, impulsivity and compulsivity can be seen as either opposing forces or overlapping constructs. Research has yet to identify a disorder driven purely by either impulsivity or compulsivity. Therefore, representing these constructs along a vertical axis (X-Y) in Figure 2 appears to be a more suitable conceptualization. This figure highlights how disorders traditionally considered impulsive or compulsive may exhibit features of both and emphasizes the overlapping and co-occurring nature of their symptoms. Moreover, (1) there is no psychiatric disorder solely characterized by pure impulsivity or pure compulsivity; (2) the same disorder may present different levels of impulsivity and compulsivity in different individuals; (3) the same disorder can exhibit varying degrees of impulsivity and compulsivity within the same individual at different times; and (4) disorders within the same diagnostic category may themselves be distributed across the impulsivity-compulsivity spectrum.

### Developmental Trajectories of Impulsivity and Compulsivity

Identifying the developmental underpinnings of impulsivity and compulsivity is crucial for understanding how these traits manifest at different life stages. Recent longitudinal evidence underscores that adolescence through early adulthood is a critical period for brain maturation, particularly within frontostriatal circuits responsible for regulating impulsive and compulsive behaviors. In a multi-year magnetic resonance imaging study, individuals with heightened impulsivity or compulsivity showed reduced myelination growth in specific frontostriatal regions, indicating that these traits may reflect altered neurodevelopmental processes extending into late adolescence.^21^ Such findings support the notion that impulsivity can present differently in younger populations—often characterized by more motor-driven or exploratory behaviors—whereas in older adolescents and adults, it may involve more complex decision-making deficits. Likewise, compulsive tendencies in adolescence may become more entrenched over time if key neurodevelopmental trajectories are disrupted. Taken together, these data emphasize that neurobiological changes underlying impulsivity and compulsivity are not fixed but evolve across developmental stages, with important implications for identifying early markers of risk and tailoring interventions accordingly.

## A Perspective on Psychiatric Disorders Through the Lens of the Impulsivity-Compulsivity Spectrum

Up to this point, an overview of the impulsivity-compulsivity spectrum, various perspectives on the topic, and the overlapping and distinct aspects of these 2 constructs have been summarized. In psychiatric clinical practice, impulsive and compulsive behaviors often play a significant role in the complaints that impair patients’ functionality and the symptoms targeted by clinicians during treatment. To better understand the prominence of these behaviors, the following sections of this article will examine specific psychiatric disorders within the framework of the impulsivity-compulsivity spectrum.^1^

### Disruptive, Impulse Control, and Conduct Disorders

Disorders in which impulsivity is a core symptom, with its destructive and negative consequences being prominent, and that are fundamentally characterized by issues in regulating emotions and behaviors, are classified under this category.^22^ Disruptive, impulse control, and conduct disorders are more prevalent in males during both childhood and adulthood. Oppositional defiant disorder and conduct disorder, which are closely related, are childhood-onset disorders within this classification.

In intermittent explosive disorder, pyromania, and kleptomania, individuals engage in destructive, problematic, and often unplanned impulsive actions following episodes of increasing tension.^23^ The tension preceding the impulsive act may escalate slowly and subtly, or, in the case of intermittent explosive disorder, it can rise rapidly in response to a mild stressor, resulting in verbal or physical aggression. In impulse control disorders, the growing tension and distress act as powerful driving forces for impulsive actions to which the individual is predisposed. Another driving force is the presence of a cue related to the pleasurable impulsive act. For instance, for someone with kleptomania, the availability of a setting conducive to theft, or for someone with pyromania, an environment suitable for starting a fire, can trigger impulsive actions. Under the influence of such triggers, an uncontrollable impulsive act is likely to occur. This act provides the individual with a sense of relief and varying degrees of pleasure or gratification. However, over time, these feelings are often replaced by guilt and remorse. During this cycle, individuals may blame and criticize themselves, but as the tension builds up again, often triggered by stressors or reminders, it becomes a driving force for another impulsive act (see Figure 1).^24^

### Obsessive-Compulsive Disorder and Related Disorders

#### Obsessive-Compulsive Disorder

The neurobiology of OCD, fundamentally aligned with the neurobiology of compulsivity, has been described in earlier sections. Functional imaging studies have identified abnormalities in the basal ganglia (particularly the caudate), cingulate cortex, and orbitofrontal cortex in OCD. Consequently, the neurobiology of OCD has been conceptualized as a dysfunction of the lateral orbitofrontal loop.^25^

As the most typical example of compulsivity, OCD provides critical insight into the impulsivity-compulsivity spectrum by examining the presence of impulsivity within its clinical presentation.^7^ Classic examples of compulsivity include repeated checking or cleaning behaviors that reduce momentary distress or perceived threat, exemplifying how anxiety relief can reinforce habitual patterns. In addition, individuals with OCD have scored significantly higher on attentional (cognitive) impulsivity scales, such as the Barratt Impulsiveness Scale. Moreover, it has been suggested that with increasing symptom severity and chronicity, certain obsessive-compulsive behaviors may become more “impulsive,” gain a pleasurable quality, and involve greater activation of ventral striatal circuits.^23^

Studies have shown that clinical factors associated with chronicity in OCD may be linked to an impulsive subtype. Additionally, it has been proposed that compulsions can sometimes occur automatically, without obsessions or anxiety symptoms, and may be driven by positive or negative reinforcement.^4^ In some individuals with OCD, compulsions may occur independently of anxiety or obsessions. In others, these compulsions are preceded by a sense of excitement or anticipation and accompanied by a vague sense of pleasure during the compulsion. One study demonstrated that deep brain stimulation of the ventral striatum (nucleus accumbens), commonly associated with impulsivity, improved OCD symptoms. Another study suggested that this improvement was independent of cognitive changes in OCD.^26^

#### Hoarding Disorder

Hoarding disorder is characterized by significant difficulty discarding or parting with possessions. It appears to overlap with obsessive-compulsive disorder (OCD) in terms of obsession-like thoughts (e.g., excessive attention and attachment to possessions), compulsive behaviors (such as collecting items), and anxiety related to discarding them. Although hoarding disorder is often conceptualized as part of the OCD spectrum, certain hoarding behaviors may arise from impulsive acquisition, suggesting a unique interplay of impulsivity and compulsivity within the same disorder. However, most individuals with hoarding disorder describe their symptoms as ego-syntonic, meaning they perceive them as aligned with their self-concept. Notably, hoarding disorder’s co-occurrence with OCD is lower than its co-occurrence with depression or other anxiety disorders. One study reported that 78.3% of individuals with hoarding disorder met the criteria for at least 1 impulse control disorder.^27^

Individuals with hoarding disorder, particularly those with excessive acquisition behaviors, have reported feelings of relief and pleasure from hoarding and acquiring items.^28^ Although evidence linking hoarding disorder to impulsivity remains limited, it is clear that the disorder does not fully align with the OCD pattern.

#### Body-Focused Repetitive Behaviors

Body-focused repetitive behaviors (BFRBs) refer to repetitive, non-rhythmic, seemingly purposeless behaviors directed at the body, such as hair-pulling (trichotillomania), skin-picking, nail-biting, and lip-biting. These behaviors are thought to serve emotion regulation functions, including reducing distress or providing pleasure. Some BFRBs, like compulsive hair-pulling, may begin impulsively but evolve into compulsive, ritualized actions. Distinguishing these nuances is crucial for tailored interventions. Previously classified under impulse control disorders, trichotillomania was reclassified as an OCD-related disorder in DSM-5.

Body-focused repetitive behaviors are considered part of the impulsivity-compulsivity spectrum, although their exact position within this spectrum remains debated.^4^ Functional connectivity studies on BFRBs have provided evidence of dysfunctional reward processing and irregularities in the reward circuitry.^29^ A recent study involving 194 participants reported that skin-picking primarily serves as an impulsive behavior that offers temporary relief from intense emotions.^30^

It is evident that the repetitive nature of BFRBs reflects a struggle with emotion regulation via behavior control. The more impulsive variants of these behaviors can manifest as self-harm (e.g., interfering with wound healing). Non-suicidal self-injury disorder is listed in DSM-5 under conditions requiring further study. The primary function of these behaviors is often to reduce negative emotions, and in more severe cases, to provide relief or even pleasure.^31^ Thus, while the more compulsive end of the spectrum is represented by BFRBs, the more impulsive end may be associated with non-suicidal self-injury.^32^ However, further research is needed in this area.

### Eating Disorders

Eating disorders exhibit a spectrum ranging from impulsivity to compulsivity. While the restrictive type of anorexia nervosa represents the compulsivity end of the spectrum, binge-purge type anorexia nervosa, bulimia nervosa, and binge eating disorder sequentially align closer to impulsive patterns.^7^^,^^33^

In binge eating disorder and bulimia nervosa, binge eating episodes are characterized by consuming large quantities of food rapidly and with low awareness, often following a period of heightened tension. During the initial moments of these episodes, individuals typically experience relief, pleasure, and satisfaction. However, guilt and regret often dominate by the end of the episode or later. In bulimia nervosa, this process is followed by compensatory behaviors such as vomiting, excessive exercise, use of laxatives or diuretics, or taking metabolism-boosting medications or herbal products. In binge eating disorder, compensatory behaviors are absent, but individuals may make promises to themselves or refrain from eating for a certain period.

From a broader perspective, binge eating episodes tend to align more with impulsive patterns, as they are often planned and driven by a desire for pleasure. In contrast, compensatory behaviors are more compulsive, serving to “reduce the distress caused by the binge episode.” Binge eating reflects a more deliberate and pleasure-seeking pattern, while compensatory behaviors are automatic and aimed at alleviating guilt and distress.

The restrictive type of anorexia nervosa, characterized by rigid control and restriction overeating and body-related behaviors, suggests a dominance of compulsivity in this disorder. In other cases, compulsive restriction may be better explained by attempts to regulate overwhelming emotions, highlighting the complexity of this presentation. These individuals are more commonly associated with OCD and obsessive-compulsive personality traits. However, it has also been proposed that the sense of control over eating provides pleasure to individuals with anorexia nervosa, suggesting a potential addiction to the behaviors associated with dietary and body control.^34^ While the reinforcement of restrictive eating patterns in anorexia nervosa can be acknowledged, directly linking these behaviors to impulsive actions is challenging.^35^

### Personality Disorders

In DSM-5, impulse control is one of the 4 domains (cognition, affectivity, interpersonal functioning, and impulse control) in which dysfunction is expected in at least 2 areas to meet the diagnostic criteria for general personality disorder. Problems with impulse control are well-known in Cluster B personality disorders, with most research focusing on antisocial personality disorder (ASPD) and borderline personality disorder (BPD).^36^ Similarly, when thinking about compulsivity, Cluster C personality disorders come to mind, but studies have predominantly examined obsessive-compulsive personality disorder (OCPD).^37^

#### Antisocial Personality Disorder

Antisocial personality disorder (ASPD) is characterized by impulsivity, alongside legal issues, deceitfulness, irresponsibility, physical aggression, disregard for societal safety, and indifference to harming or exploiting others.^6^ Research consistently links ASPD with high scores on all dimensions of impulsivity compared to healthy controls. Aggressive behavior in ASPD is studied in 2 forms: reactive aggression, which arises as a response to emotional stimuli, and proactive aggression, which is goal-oriented and independent of emotion. Both forms of aggression are elevated in ASPD compared to healthy controls. Additionally, aggression in ASPD is more pronounced than in other personality disorders. Individuals with ASPD also perform poorly on tasks related to response inhibition and delay of gratification, reflecting impairments in higher cortical functions of impulsivity.^36^

A biological marker associated with aggression, monoamine oxidase A, has been found at low levels in cortical and subcortical regions of individuals with ASPD. Lower monoamine oxidase A levels in the ventral striatum and dorsal putamen have been negatively correlated with delay-of-gratification performance and self-reported impulsivity scores in ASPD.^38^

Although high impulsivity is evident in ASPD, symptoms are often explained through the lens of aggression. However, aggression in ASPD is not solely a consequence of impulsivity, as impulsivity in disorders like BPD does not typically result in the same level of aggression. A core symptom of ASPD is impaired emotional empathy.^39^ Social norms, personal rights, and moral conscience—factors that usually suppress aggression—are ineffective in ASPD. These individuals have strong driving forces for aggression and harm (high impulsivity) and weak inhibitory forces (low empathy and moral conscience).

A study examining the relationship between impulsivity and compulsivity with personality traits found that higher impulsivity was associated with increased symptoms of ASPD and ADHD, as well as greater suicidal tendencies. On the other hand, increased compulsivity was linked to higher levels of gambling, problematic internet use, substance use disorders, and OCPD traits.^8^ Therefore, although scientific studies on compulsivity in ASPD are not widespread, it is possible that substance use disorders and other behavioral addictions co-occurring with ASPD may involve compulsive behaviors.

#### Borderline Personality Disorder

Borderline personality disorder (BPD) is a personality disorder characterized by impulsivity as a diagnostic criterion, alongside interpersonal instability, identity disturbances, anger outbursts, chronic feelings of emptiness, self-harm, and suicidal behaviors. A history of childhood trauma is common in individuals with BPD.^6^ These individuals exhibit impulsivity components such as positive urgency (acting rashly to achieve pleasure), lack of premeditation, and sensation-seeking through behaviors like unprotected sexual activity, substance abuse, excessive spending, and disordered eating. Additionally, BPD individuals may engage in self-harm or suicidal behaviors to escape heightened negative emotions or feelings of emptiness. These behaviors exemplify negative urgency (acting rashly to escape distress), though other impulsivity components may also contribute. Studies have identified objective cognitive impairments in BPD, such as poor performance in delay-of-gratification and response-inhibition tasks.^36^

While BPD is strongly associated with impulsivity, suicidal thoughts in BPD cannot always be reduced to impulsive patterns triggered by external stimuli.^40^ Many BPD individuals describe suicidal thoughts as chronic, even ego-syntonic, suggesting that such thoughts could be obsessive in nature. Similarly, self-harm behaviors, often aimed at alleviating negative emotions, may reflect compulsive tendencies. A study found that BPD individuals who engaged in self-harm exhibited higher levels of obsessive-compulsive symptoms compared to those who did not.^41^ Behaviors like substance abuse, casual sexual encounters, and disordered eating patterns may become repetitive and compulsive patterns aimed at reducing tension and distress. In personality disorders with externalizing symptoms, like ASPD, impulsivity manifests as aggression and anger. However, in disorders like BPD, where internalizing symptoms predominate, impulsivity often appears as self-harm or suicidal behavior.^42^

#### Obsessive-Compulsive Personality Disorder

Obsessive-compulsive personality disorder (OCPD) is characterized by a strong adherence to routines, excessive preoccupation with details, a desire for clarity (intolerance of uncertainty), and avoidance of novelty or risk. These features make OCPD the most prototypical example of compulsivity. Intolerance of uncertainty and the desire for clarity in OCPD overlap with the negative urgency component of impulsivity, though this component is inherently tied to compulsivity, as previously discussed.

Obsessive-compulsive personality disorder (and possibly the restrictive type of anorexia nervosa) represents disorders most closely aligned with compulsivity. However, considering that compulsivity and impulsivity can act as triggers for one another, impulsive behaviors may also emerge in OCPD.^37^ The National Epidemiologic Survey on Alcohol and Related Conditions found significant associations between OCPD and various impulsivity-related disorders, such as substance use disorders and ADHD.^43^ Moreover, compared to healthy controls, individuals with OCPD report higher levels of negative affect, persistent anger, emotional intensity, and difficulties in emotion regulation, which are often associated with impulsivity.^44^ Taken together, it is plausible that some impulsive elements are present in at least a subset of individuals with OCPD.

### Substance-Related and Addictive Disorders

Impulsivity is a fundamental process in the development of both substance use and behavioral addictions, although its severity varies across different types of addiction.^17^ Conversely, many individuals with addictive behaviors exhibit neurocognitive deficits in motivational and self-regulatory circuits, which increase their risk of developing or maintaining substance or behavioral addictions. The complex causality of addictive behaviors and the diversity of study designs in this area have made it difficult to directly link any specific substance-related or behavioral addiction exclusively to impulsivity or compulsivity.^45^ The tendencies to act “impulsively” and “compulsively” are 2 core processes that contribute to the development of addictive behaviors to varying degrees. Understanding how the distinct neurocognitive functions underlying impulsivity and compulsivity are shared or specific to addictive behaviors from a transdiagnostic perspective may improve the understanding of the cognitive mechanisms driving addictions.^46^ Recent evidence suggests that deficits in shift reversal and inhibitory control, along with elevated compulsive traits, may serve as markers of familial vulnerability in the development of substance use disorders, particularly heroin dependence. Such findings highlight the importance of examining compulsivity-related neurocognitive impairments not only in individuals with addiction but also in their unaffected siblings, providing a broader perspective on transdiagnostic mechanisms and hereditary predispositions.^47^

Substance misuse or encounters with substances often evoke thoughts of impulsive actions among clinicians. This is likely because such encounters and experimentation are associated with neurocognitive deficits related to impulsivity, such as sensation-seeking, positive urgency, and response inhibition deficits. However, “addiction” represents a clinical syndrome characterized by persistence, impairment across multiple domains of functioning, and a predominance of negative emotions. Substance use in response to withdrawal (reducing distress), failed attempts to quit (ego-dystonic experiences), and excessive time spent obtaining or using substances (attentional fixation and cognitive inflexibility) align with compulsive features. Meanwhile, craving (expectation of pleasure) and continued use despite negative consequences are more reflective of impulsive traits. Response inhibition serves as a shared mechanism in the inability to control both positively and negatively reinforced behaviors.

Coping with negative emotions is a core component of the addiction process. For at least some individuals, what begins as an impulsive behavior progresses into a stage where compulsive behaviors become inevitable. For example, an individual who occasionally consumes alcohol socially at home may increase their alcohol use to alleviate structural or acute fears and anxiety, ultimately developing a dependency. This progression suggests that the addiction process may take on a predominantly compulsive trajectory. Moreover, findings from recent research indicate that compulsivity-related impairments, such as deficits in set-shifting and reversal learning, are not only present in individuals with substance use disorders but also in their unaffected siblings, underscoring the potential role of hereditary vulnerabilities in driving these compulsive patterns.^47^

#### Gambling Disorder

Gambling disorder is classified under “Substance-Related and Addictive Disorders” in DSM-5 as a “Non-Substance-Related Disorder”.^6^ Positioned at the crossroads of the impulsivity-compulsivity spectrum, gambling disorder includes diagnostic criteria aligned with impulsivity (gambling to achieve desired pleasure) and compulsivity (gambling to reduce negative emotions). Two recent meta-analyses provide support for this duality: 1 highlights executive function deficits related to impulsivity, while the other emphasizes deficits related to compulsivity in gambling disorder.^48^^,^^49^ Similarly, compulsive buying disorder shares its place on the impulsivity-compulsivity spectrum with gambling disorder, but findings remain inconclusive as to which end of the spectrum it aligns more closely with.^50^

### Neurodevelopmental Disorders

Neurodevelopmental disorders are conditions that manifest in childhood and are characterized by pervasive and varying issues in inhibitory control, with executive dysfunction being a core feature.^51^ These widespread executive function deficits lead to numerous adverse outcomes throughout life, including comorbid psychiatric symptoms and disorders, difficulties in social relationships, academic and occupational challenges, and additional physical health problems. At least two-thirds of adults with ADHD or autism spectrum disorder (ASD) have at least 1 psychiatric comorbidity. The failure to adequately assess neurodevelopmental disorders in adulthood, particularly in complex and challenging cases, may result in missing key pieces of the diagnostic puzzle. Attention-deficit hyperactivity disorder and autism are often seen as residing at opposing ends of the impulsivity-compulsivity spectrum based on their diagnostic criteria and clinical presentations.

#### Attention-Deficit Hyperactivity Disorder

Attention-deficit hyperactivity disorder (ADHD), although categorized into 3 symptom clusters—attention-deficit, hyperactivity, and impulsivity—all of its symptoms are inherently linked to impulsivity. The pervasive and developmental impulse control problems in ADHD have led to its conceptualization as a frontal cortex dysfunction and a clear example of a top-down inhibition deficit. The diagnostic criteria for hyperactivity/impulsivity in ADHD, such as difficulty waiting in line, interrupting others, blurting out answers, restlessness, and inability to stay seated, are closely associated with the urgency components of impulsivity (positive or negative). Behaviors such as excessive talking, constant fidgeting, running about, or appearing “driven by a motor” are primarily linked to inner hyperactivity. These symptoms are not limited to motor activities; they also include cognitive hyperactivity, such as an inability to focus on a single thought, frequent topic-switching, difficulty sustaining tasks, and rapid, competing, or racing thoughts. This pattern, often observed as “mental hyperactivity,” has been described as “excessive mind-wandering” and is proposed as one of the most distinctive features of adult ADHD.^52^

The diagnostic criteria for ADHD are primarily related to physical/mental hyperactivity and urgency, which do not completely align with impulsivity characterized by sensation-seeking, risk-taking, or acting without regard for consequences. However, increased internal energy and reduced inhibitory control contribute to impulsive actions and an elevated risk of psychiatric disorders involving impulse control. Furthermore, these symptoms inevitably lead to widespread executive function challenges, such as disorganization, poor time management, and difficulties with planning. Compulsive behaviors in ADHD should be addressed during diagnostic and individual evaluations.

#### Autism Spectrum Disorder

Autism spectrum disorder is a neurodevelopmental disorder characterized by impairments in social communication and interaction, alongside restricted and repetitive patterns of interests and behaviors. The restricted/repetitive behavior domain in ASD includes adherence to routines, insistence on sameness, intense focus on restricted interests, altered responses to external stimuli, and stereotyped behaviors, all of which closely overlap with compulsive patterns.^53^ This symptom pattern may lower the threshold for inhibition, increasing the likelihood of planned or stereotyped aggressive behaviors. The most frequently targeted symptoms in pharmacological treatments for individuals with ASD are impulsive and compulsive behavioral problems. These behaviors are critical considerations for clinical interventions aimed at improving quality of life and reducing functional impairment.

### Bipolar Disorder

The manic phase of bipolar disorder is characterized by increased energy and activity (e.g., excessive talking, flight of ideas, heightened energy levels) and a reduced perception of risk, resulting in the most severe manifestations of impulsivity during this period. Patients in the manic phase report high levels of impulsivity on self-report measures and demonstrate impaired response inhibition, delay of gratification, and attentional difficulties on executive function tests. While executive function impairments tend to normalize during depressive and euthymic phases, self-reported impulsivity scores remain elevated in the euthymic phase compared to healthy controls. This suggests that structural impulsivity traits, rather than solely episodic features, may serve as a vulnerability factor that facilitates the onset of manic episodes in individuals with bipolar disorder.^54^

## Discussion

Although this is a narrative review rather than a systematic review, a keyword-based literature search (PubMed, PsycINFO, Web of Science) was conducted for English-language, peer-reviewed articles published from 2000 to 2023. Keywords included “impulsivity,” “compulsivity,” “psychiatric disorders,” “dimensional approach,” and specific diagnostic terms (e.g., “ADHD,” “OCD,” “substance use disorders”). Articles addressing theoretical frameworks, clinical presentations, or neurobiological underpinnings were selected, allowing a broad synthesis of the impulsivity-compulsivity spectrum without the constraints of a formal systematic review protocol.

This review highlights the overlapping and dynamic nature of impulsivity and compulsivity as dimensions that co-occur across a wide range of psychiatric disorders. Impulsivity is prominently observed in disorders such as ADHD, impulse control disorders, and manic episodes of bipolar disorder, while compulsivity characterizes conditions like OCD, OCPD, and restrictive anorexia nervosa. However, many disorders, including gambling disorder, substance use disorders, and BPD, demonstrate features of both impulsivity and compulsivity.^55^ The findings underscore the necessity of adopting a dimensional framework to better capture the shared and distinct mechanisms underlying these constructs and their contributions to psychiatric symptomatology.^21^

It is evident that impulsivity and compulsivity do not represent 2 opposing and entirely separate extremes. While their behavioral patterns and the emotional regulation models they serve may differ, both constructs share underlying difficulties in perseverance and inhibitory control.^7^^,^^8^ They frequently co-occur at both the symptom and diagnostic levels. Just as there are no psychiatric disorders solely defined by either impulsivity or compulsivity, the dominance of 1 often makes the presence of the other inevitable. For example, a behavior such as pathological gambling may appear the same externally, but 1 individual may engage in it impulsively, while another exhibits a more compulsive pattern. Similarly, the same individual might display behavior like compulsive shopping in a more impulsive manner at times and in a more compulsive manner at others. In clinical practice, it is essential to assess patients both diagnostically and individually.

### Clinical Implications

A dimensional framework for impulsivity and compulsivity helps clinicians identify the severity and primary drivers of maladaptive behaviors more precisely than purely categorical diagnoses. Screening tools such as the Barratt Impulsiveness Scale and the Urgency, (lack of) Premeditation, (lack of) Perseverance, and Sensation Seeking (UPPS) Impulsive Behavior Scale are widely used to measure impulsivity, while the Yale-Brown Obsessive Compulsive Scale is particularly useful for assessing compulsive traits. Individuals who exhibit high impulsivity may benefit from techniques that target impulse control and distress tolerance—such as motivational interviewing, self-monitoring strategies, contingency management (often used for attention-deficit/hyperactivity disorder), and dialectical behavior therapy. In contrast, those with pronounced compulsive symptoms often respond better to cognitive-behavioral approaches like exposure and response prevention, which specifically tackles the anxiety-driven habits seen in disorders such as OCD.^17^ In addition, mindfulness-based interventions have gained traction for both impulsivity and compulsivity, as they focus on enhancing self-regulation, emotional awareness, and executive function. Pharmacotherapy can also be tailored to these dimensions: selective serotonin reuptake inhibitors are typically prescribed for compulsivity, while stimulants may be indicated for impulsivity (particularly in ADHD). The combined use of these medications should be carefully considered in patients who present with both impulsive and compulsive behaviors.

Given that impulsive and compulsive behaviors often overlap with conditions like BPD, substance use disorders, or bipolar disorder, multi-modal treatment approaches are frequently necessary.^55^ Notably, high impulsivity has been associated with poorer treatment adherence (e.g., in BPD), while entrenched compulsive patterns can perpetuate symptom severity, underscoring the need for interventions that address long-term outcomes and relapse prevention. While this review outlines various interventions, study methods, and populations vary widely, raising concerns about measurement consistency and selection bias. As a narrative review, the quality or design of each study was not systematically evaluated. A more critical appraisal of these factors would help solidify the conclusions and guide future research into dimensional, patient-centered treatment strategies.

### Future Directions

Although categorical diagnostic systems have historically guided mental healthcare, an expanding body of evidence, improved assessment tools, and patient-centered priorities underscore a shift toward dimensional frameworks. In this context, the Research Domain Criteria project initiated by the US National Institute of Mental Health advocates evaluating constructs such as attention, response inhibition, and reward responsiveness dimensionally—an approach particularly applicable to impulsivity and compulsivity.^56^ Large-scale, longitudinal studies tracing these traits from adolescence into adulthood would clarify their developmental trajectories, especially when combined with genetic polymorphism data and environmental factors (e.g., childhood adversity). Advances in neuroimaging—particularly focused on frontostriatal circuits—are equally crucial for identifying shared or divergent endophenotypes underlying impulsive and compulsive manifestations. By refining dimensional assessment tools, researchers can streamline preventive interventions and patient-specific care. Finally, emerging digital and mobile health applications offer real-time monitoring of impulsive and compulsive tendencies, enabling targeted, immediate interventions that further enhance clinical outcomes.

## Conclusion

Impulsivity and compulsivity share fundamental difficulties in perseverance and inhibitory control, often driven by the urgent pursuit of pleasure or the urgent need to relieve distress. These constructs also converge on overlapping neurobiological circuits—such as those involved in reward processing and top-down regulation—highlighting the importance of a dimensional perspective that captures both their commonalities and distinctions. Recognizing the interplay of impulsivity and compulsivity can help clinicians and researchers more effectively assess and address the spectrum of behaviors observed in psychiatric disorders. By moving beyond purely categorical diagnoses, individualized interventions can be developed to target both the shared and disorder-specific mechanisms underlying dysfunctional impulsive and compulsive actions. Future research should emphasize developing dimensional assessment tools and interventions that address the overlapping features of impulsivity and compulsivity, ultimately refining diagnostic precision and enhancing individualized treatment approaches across psychiatric disorders.

## Data Availability

The data that support the findings of this study are available on request from the corresponding author.

## References

[1] Moeller FG Barratt ES Dougherty DM Schmitz JM Swann AC. Psychiatric aspects of impulsivity. Am J Psychiatry. 2001;158(11):1783 1793. (doi: 10.1176/appi.ajp.158.11.1783) 11691682

[2] Eysenck HJ Eysenck MW. Pers Individ Dif. 1985.

[3] Cloninger CR Svrakic DM Przybeck TR. A psychobiological model of temperament and character. Arch Gen Psychiatry. 1993;50(12):975 990. (doi: 10.1001/archpsyc.1993.01820240059008) 8250684

[4] Robbins TW Gillan CM Smith DG de Wit S Ersche KD. Neurocognitive endophenotypes of impulsivity and compulsivity: towards dimensional psychiatry. Trends Cogn Sci. 2012;16(1):81 91. (doi: 10.1016/j.tics.2011.11.009) 22155014

[5] Hollander E Wong CM. Obsessive-compulsive spectrum disorders. J Clin Psychiatry. 1995;56(Suppl 4):53 55. 7713863

[6] American Psychiatric Association. Diagnostic and Statistical Manual of Mental Disorders. 5th ed. Washington, DC: American Psychiatric Association; 2022.

[7] Robbins TW Banca P Belin D. From compulsivity to compulsion: the neural basis of compulsive disorders. Nat Rev Neurosci. 2024;25(5):313 333. (doi: 10.1038/s41583-024-00807-z) 38594324

[8] Chamberlain SR Stochl J Redden SA Grant JE. Latent traits of impulsivity and compulsivity: toward dimensional psychiatry. Psychol Med. 2018;48(5):810 821. (doi: 10.1017/S0033291717002185) 28805173 PMC5699644

[9] Fineberg NA Potenza MN Chamberlain SR Probing compulsive and impulsive behaviors, from animal models to endophenotypes: a narrative review. Neuropsychopharmacology. 2010;35(3):591 604. (doi: 10.1038/npp.2009.185) 19940844 PMC3055606

[10] Gruner P Pittenger C. Cognitive inflexibility in obsessive-compulsive Disorder. Neuroscience. 2017;345:243 255. (doi: 10.1016/j.neuroscience.2016.07.030) 27491478 PMC5288350

[11] Grant JE Kim SW. Brain circuitry of compulsivity and impulsivity. CNS Spectr. 2014;19(1):21 27. (doi: 10.1017/S109285291300028X) 23659364

[12] Dalley JW Everitt BJ Robbins TW. Impulsivity, compulsivity, and top-down cognitive control. Neuron. 2011;69(4):680 694. (doi: 10.1016/j.neuron.2011.01.020) 21338879

[13] Kable JW Glimcher PW. An “as soon as possible” effect in human intertemporal decision making: behavioral evidence and neural mechanisms. J Neurophysiol. 2010;103(5):2513 2531. (doi: 10.1152/jn.00177.2009) 20181737 PMC2867580

[14] Brennan BP Rauch SL Jensen JE Pope HG Jr. A critical review of magnetic resonance spectroscopy studies of obsessive-compulsive disorder. Biol Psychiatry. 2013;73(1):24 31. (doi: 10.1016/j.biopsych.2012.06.023) 22831979 PMC3504626

[15] Ting JT Feng G. Neurobiology of obsessive–compulsive disorder: insights into neural circuitry dysfunction through mouse genetics. Curr Opin Neurobiol. 2011;21(6):842 848. (doi: 10.1016/j.conb.2011.04.010) 21605970 PMC3192923

[16] Harrison BJ Soriano-Mas C Pujol J Altered corticostriatal functional connectivity in obsessive-compulsive disorder. Arch Gen Psychiatry. 2009;66(11):1189 1200. (doi: 10.1001/archgenpsychiatry.2009.152) 19884607

[17] Aguilar-Yamuza B Trenados Y Herruzo C Pino MJ Herruzo J. A systematic review of treatment for impulsivity and compulsivity. Systematic review. Front Psychiatry. 2024;15:1430409. (doi: 10.3389/fpsyt.2024.1430409) PMC11465090 39391084

[18] Grant JE Donahue CB Odlaug BL Kim SW Miller MJ Petry NM. Imaginal desensitisation plus motivational interviewing for pathological gambling: randomised controlled trial. Br J Psychiatry. 2009;195(3):266 267. (doi: 10.1192/bjp.bp.108.062414) 19721120 PMC2801822

[19] Whiteside SP Lynam DR. The Five Factor Model and impulsivity: using a structural model of personality to understand impulsivity. Pers Individ Dif. 2001;30(4):669 689. (doi: 10.1016/S0191-8869(00)00064-7)

[20] Grassi G Pallanti S Righi L Think twice: impulsivity and decision making in obsessive-compulsive disorder. J Behav Addict. 2015;4(4):263 272. (doi: 10.1556/2006.4.2015.039) 26690621 PMC4712760

[21] Ziegler G Hauser TU Moutoussis M Compulsivity and impulsivity traits linked to attenuated developmental frontostriatal myelination trajectories. Nat Neurosci. 2019;22(6):992 999. (doi: 10.1038/s41593-019-0394-3) 31086316 PMC7610393

[22] Robbins TW Curran HV de Wit H. Special issue on impulsivity and compulsivity. Psychopharmacology. 2012;219(2):251 252. (doi: 10.1007/s00213-011-2584-x) 22124671

[23] Fontenelle LF Oostermeijer S Harrison BJ Pantelis C Yücel M. Obsessive-compulsive disorder, impulse control disorders and drug addiction: Common features and potential treatments. Drugs. 2011;71(7):827 840. (doi: 10.2165/11591790-000000000-00000) 21568361

[24] Kwako LE Koob GF. Neuroclinical framework for the role of stress in addiction. Chronic Stress (Thousand Oaks). 2017;1:2470547017698140. (doi: 10.1177/2470547017698140) 28653044 PMC5482275

[25] Del Casale A Kotzalidis GD Rapinesi C Functional neuroimaging in obsessive-compulsive disorder. Neuropsychobiology. 2011;64(2):61 85. (doi: 10.1159/000325223) 21701225

[26] Denys D Mantione M Figee M Deep brain stimulation of the nucleus accumbens for treatment-refractory obsessive-compulsive disorder. Arch Gen Psychiatry. 2010;67(10):1061 1068. (doi: 10.1001/archgenpsychiatry.2010.122) 20921122

[27] Frost RO Steketee G Tolin DF. Comorbidity in hoarding disorder. Focus. 2015;13(2):244 251. (doi: 10.1176/appi.focus.130218) PMC3188689 21770000

[28] Rasmussen JL Brown TA Steketee GS Barlow DH. Impulsivity in hoarding. J Obsessive Compulsive Relat Disord. 2013;2(2):183 191. (doi: 10.1016/j.jocrd.2013.02.004)

[29] Grant JE Peris TS Ricketts EJ Reward processing in trichotillomania and skin picking disorder. Brain Imaging Behav. 2022;16(2):547 556. (doi: 10.1007/s11682-021-00533-5) 34410609 PMC7614803

[30] Schienle A Zorjan S Übel S Wabnegger A. Prediction of automatic and focused skin picking based on trait disgust and emotion dysregulation. J Obsessive Compulsive Relat Disord. 2018;16:1 5. (doi: 10.1016/j.jocrd.2017.10.006)

[31] Taylor PJ Jomar K Dhingra K Forrester R Shahmalak U Dickson JM. A meta-analysis of the prevalence of different functions of non-suicidal self-injury. J Affect Disord. 2018;227:759 769. (doi: 10.1016/j.jad.2017.11.073) 29689691

[32] Kandeger A Uygur OF Ataslar EY Cınar F Selvi Y. A pilot study examining hemomania behaviors in psychiatry outpatients engaged with nonsuicidal self-injury. Brain Behav. 2024;14(4):e3475. (doi: 10.1002/brb3.3475) 38594228 PMC11004038

[33] Lavender JM Goodman EL Culbert KM Facets of impulsivity and compulsivity in women with anorexia nervosa. Eur Eat Disord Rev. 2017;25(4):309 313. (doi: 10.1002/erv.2516) 28387426 PMC7654514

[34] Godier LR Park RJ. Compulsivity in anorexia nervosa: a transdiagnostic concept. Front Psychol. 2014;5:778. (doi: 10.3389/fpsyg.2014.00778) 25101036 PMC4101893

[35] Treasure J Zipfel S Micali N Anorexia nervosa. Nat Rev Dis Primers. 2015;1(1):15074. (doi: 10.1038/nrdp.2015.74) 27189821

[36] Turner D Sebastian A Tüscher O. Impulsivity and cluster B personality disorders. Curr Psychiatry Rep. 2017;19(3):15. (doi: 10.1007/s11920-017-0768-8) 28251591

[37] Grant JE Chamberlain SR. Obsessive compulsive personality traits: understanding the chain of pathogenesis from health to disease. J Psychiatr Res. 2019;116:69 73. (doi: 10.1016/j.jpsychires.2019.06.003) 31202047 PMC7099944

[38] Kolla NJ Dunlop K Downar J Association of ventral striatum monoamine oxidase-A binding and functional connectivity in antisocial personality disorder with high impulsivity: a positron emission tomography and functional magnetic resonance imaging study. Eur Neuropsychopharmacol. 2016;26(4):777 786. (doi: 10.1016/j.euroneuro.2015.12.030) 26908392

[39] Velotti P Garofalo C Dimaggio G Fonagy P. Mindfulness, alexithymia, and empathy moderate relations between trait aggression and antisocial personality disorder traits. Mindfulness. 2019;10(6):1082 1090. (doi: 10.1007/s12671-018-1048-3)

[40] Paris J. Chronic suicidality among patients with borderline personality disorder. Psychiatr Serv. 2002;53(6):738 742. (doi: 10.1176/appi.ps.53.6.738) 12045312

[41] McKay D Kulchycky S Danyko S. Borderline personality and obsessive-compulsive symptoms. J Pers Disord. 2000;14(1):57 63. (doi: 10.1521/pedi.2000.14.1.57) 10746205

[42] Hahn AM Simons RM Tirabassi CK. Five factors of impulsivity: unique pathways to borderline and antisocial personality features and subsequent alcohol problems. Pers Individ Dif. 2016;99:313 319. (doi: 10.1016/j.paid.2016.05.035) 32123459 PMC7050995

[43] Grant JE Mooney ME Kushner MG. Prevalence, correlates, and comorbidity of DSM-IV obsessive-compulsive personality disorder: results from the National Epidemiologic Survey on alcohol and Related Conditions. J Psychiatr Res. 2012;46(4):469 475. (doi: 10.1016/j.jpsychires.2012.01.009) 22257387

[44] Steenkamp MM Suvak MK Dickstein BD Shea MT Litz BT. Emotional functioning in obsessive-compulsive personality disorder: comparison to borderline personality disorder and healthy controls. J Pers Disord. 2015;29(6):794 808. (doi: 10.1521/pedi_2014_28_174) 25562536

[45] Tiego J Oostermeijer S Prochazkova L Overlapping dimensional phenotypes of impulsivity and compulsivity explain co-occurrence of addictive and related behaviors. CNS Spectr. 2019;24(4):426 440. (doi: 10.1017/S1092852918001244) 30458896

[46] Lee RSC Hoppenbrouwers S Franken I. A systematic meta-review of impulsivity and compulsivity in addictive behaviors. Neuropsychol Rev. 2019;29(1):14 26. (doi: 10.1007/s11065-019-09402-x) 30927147

[47] Yan W-S Liu S-J Zheng D-H. Compulsivity and inhibitory control deficits in abstinent individuals with heroin addiction and their biological siblings compared with unrelated healthy control participants. Biol Psychiatry Cogn Neurosci Neuroimaging. 2024;9(2):196 206. (doi: 10.1016/j.bpsc.2023.11.002) 37995811

[48] Ioannidis K Hook R Wickham K Grant JE Chamberlain SR. Impulsivity in Gambling Disorder and problem gambling: a meta-analysis. Neuropsychopharmacology. 2019;44(8):1354 1361. (doi: 10.1038/s41386-019-0393-9) 30986818 PMC6588525

[49] van Timmeren T Daams JG van Holst RJ Goudriaan AE. Compulsivity-related neurocognitive performance deficits in gambling disorder: a systematic review and meta-analysis. Neurosci Biobehav Rev. 2018;84:204 217. (doi: 10.1016/j.neubiorev.2017.11.022) 29203423

[50] Montemarano V Kim HS Antony MM. A comparison of buying disorder to addictive and obsessive–compulsive disorders on impulsivity, compulsivity, and reward processing: a narrative review. Curr Psychol. 2024;43(10):9336 9354. (doi: 10.1007/s12144-023-05040-y)

[51] Mirabella G. Inhibitory control and impulsive responses in neurodevelopmental disorders. Dev Med Child Neurol. 2021;63(5):520 526. (doi: 10.1111/dmcn.14778) 33340369

[52] Kooij JJS Bijlenga D Salerno L Updated European Consensus Statement on diagnosis and treatment of adult ADHD. Eur Psychiatry. 2019;56(1):14 34. (doi: 10.1016/j.eurpsy.2018.11.001) 30453134

[53] Pazuniak M Pekrul SR. Obsessive–compulsive disorder in autism spectrum disorder across the lifespan. Child Adolesc Psychiatr Clin N Am. 2020;29(2):419 432. (doi: 10.1016/j.chc.2019.12.003) 32169271

[54] Newman AL Meyer TD. Impulsivity: present during euthymia in bipolar disorder? - a systematic review. Int J Bipolar Disord. 2014;2(1):2. (doi: 10.1186/2194-7511-2-2) 25960939 PMC4424222

[55] Crisp ZC Grant JE. Impulsivity across psychiatric disorders in young adults. Compr Psychiatry. 2024;130:152449. (doi: 10.1016/j.comppsych.2023.152449) 38184857

[56] Insel T Cuthbert B Garvey M Research domain criteria (RDoC): toward a new classification framework for research on mental disorders. Am J Psychiatry. 2010;167(7):748 751. (doi: 10.1176/appi.ajp.2010.09091379) 20595427

